# Idiopathic Bilateral Suprachoroidal Haemorrhage: A Rare Case Presentation

**DOI:** 10.1155/2017/4234238

**Published:** 2017-06-28

**Authors:** Komal Saluja, Mayuresh Naik, Rajshekhar Vemparala, Anuj Mehta

**Affiliations:** Department of Ophthalmology, VMMC & Safdarjung Hospital, Ring Road, Ansari Nagar, New Delhi 110029, India

## Abstract

55-year-old male presented with sudden onset painful diminution of vision in both eyes. On local examination, his visual acuity was FC at 2 metres in right eye and FC at 1 m in left eye. The IOP in right eye was 46 mm Hg and 44 mm Hg in left eye. The patient was admitted and started on injection mannitol, oral syrup glycerol, and oral acetazolamide. Locally, timolol maleate and brimonidine were also started. The next day, his IOP was 17 mm Hg bilaterally but his visual acuity deteriorated to FC 1 m in right eye and hand movement in left eye with inaccurate projection of rays in both eyes. USG B-scan was performed which revealed bilateral choroidal detachment. The echotexture of fluid was suggestive of haemorrhage. As the IOP was controlled, systemic hyperosmotic/antiglaucoma agents were withdrawn in stepwise fashion over next two days. The patient was started on oral prednisolone. At 2 weeks, the visual acuity in both eyes was only perception of light, with inaccurate PR. IOP was 10 mm Hg in both eyes. USG B-scan revealed resorption of the hemorrhage, with partial resolution of the choroidal detachment. The final BCVA was 6/18 and 6/12 in right and left eye.

## 1. Introduction

Suprachoroidal haemorrhage is a rare but devastating complication of intraocular surgery. Spontaneous suprachoroidal haemorrhage is an even rarer disease. Only few cases have been reported in literature. Most of these cases were associated with deranged coagulation profile due to either some disease or drugs. We are reporting a case of spontaneous bilateral suprachoroidal haemorrhage which presented with bilateral angle closure glaucoma without any associated coagulation disorders.

## 2. Case Report

A 55-year-old male, known smoker and alcoholic, presented to our emergency, with sudden onset pain and diminution of vision of both eyes. His medical history was unremarkable with no history of any systemic or ocular disease.

On examination, his vitals were stable, but icterus was noted. On local examination, his visual acuity was counting fingers (CF) at 2 metres in right eye and CF at 1 m in left eye. The intraocular pressure (IOP) in right eye was 46 mmHg and 44 mmHg in left eye. On slit lamp biomicroscopy, epithelial corneal edema was noted with shallow AC in both eyes. Pupils were mid-dilated and fixed in both eyes. Fundus could not be evaluated bilaterally due to media haze.

The patient was admitted and started on injection mannitol 20% (1 g/kg body wt) 8-hourly, oral syrup glycerol 50% (1 g/kg body wt) thrice a day, and oral acetazolamide, tablet 250 mg, thrice daily, with potassium supplement. Locally, timolol maleate 0.50% eye drop twice daily and brimonidine 0.2% eye drop twice daily were also started.

**Figure 1:**
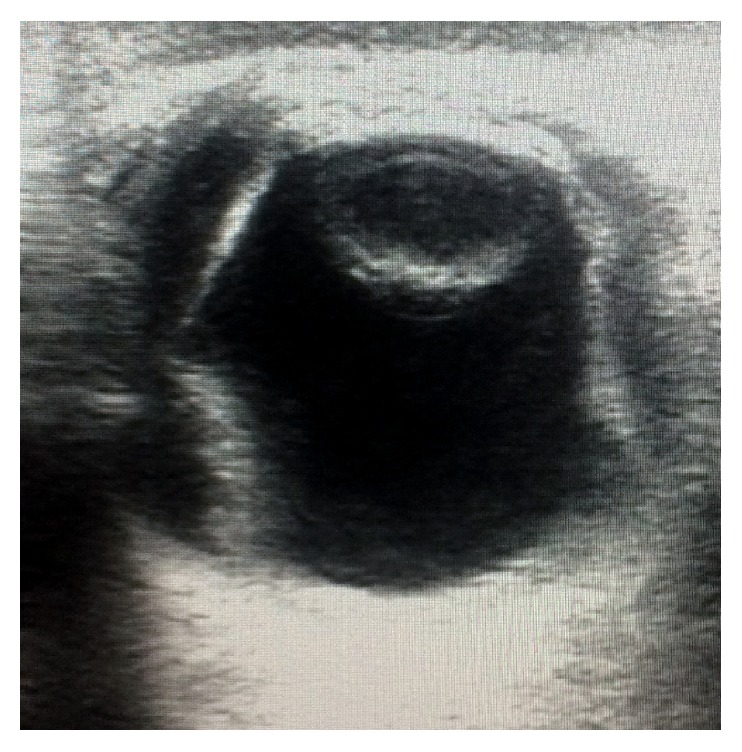
USG B-scan showing choroidal detachment with moderate intensity echoes in suprachoroidal space.

**Figure 2:**
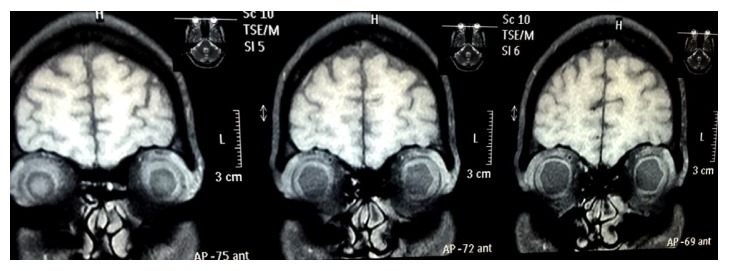
Coronal T1 weighted MRI of head and orbit showing bilateral choroidal detachment with bilateral suprachoroidal haemorrhage.

**Figure 3:**
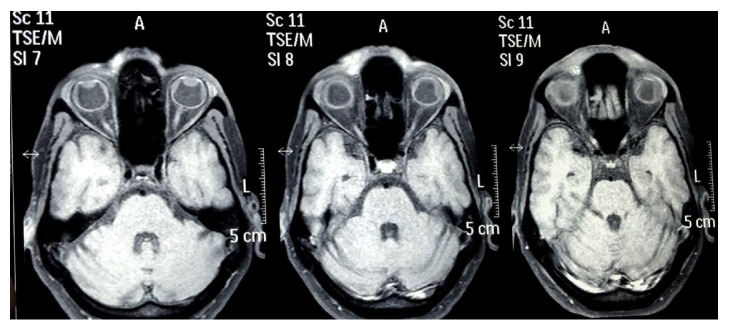
Axial T1 weighted MRI of head and orbit showing bilateral choroidal detachment with bilateral suprachoroidal haemorrhage.

The patient was reevaluated the next day. His IOP was 17 mmHg bilaterally but his visual acuity deteriorated to CF at 1 m in right eye and hand movement in left eye with inaccurate projection of rays in both eyes. Anterior chamber was still shallow, more in centre than periphery, but there was no lens-corneal touch. Pigment deposition was noted with deposition over lens and corneal endothelium. Pupil was still mid-dilated and fixed. Fundus examination revealed yellow reflex and details could not be seen. USG B-scan was performed which revealed bilateral choroidal detachment and fluid collection in suprachoroidal space. The echotexture of fluid was suggestive of haemorrhage (Figure 1). The complete blood count and coagulation tests revealed no abnormality (INR 1.03). Liver function tests showed raised bilirubin levels (total 11.8 mg/dl, direct bilirubin 9.4 mg/dl). Liver enzymes SGOT and SGPT were also raised; however GGT levels were normal. HbS Ag and HbC Ag were nonreactive. MRI orbit done showed bilateral suprachoroidal haemorrhage (Figures 2 and 3). CECT abdomen showed a hemangioma in liver lobe IV, which was reported to be an incidental finding. As the IOP was controlled, systemic hyperosmotic/antiglaucoma agents were withdrawn in stepwise fashion over the next two days. The patient was started on oral prednisolone (1 mg/kg body wt) once a day.

At 2 weeks, the visual acuity in both eyes was only perception of light, with inaccurate PR. IOP was 10 mmHg in both eyes and fundus examination still showed yellowish reflex. USG B-scan revealed resorption of the haemorrhage, with partial resolution of the choroidal detachment. Liver enzymes and total bilirubin returned to normal levels. Tab prednisolone was tapered over a period of the next two weeks and poor visual prognosis explained to the patient. At two months' follow-up, the patient presented with total cataract bilaterally, with anechoic USG B-scan and normal MRI orbit. Bilateral cataract surgery was performed with intraocular lens implantation. Fundus examination after cataract surgery revealed normal optic disc and macula, well attached retina, and normal choroid. The final best corrected visual acuity was 6/18 and 6/12 in right and left eye.

## 3. Discussion

The suprachoroidal haemorrhage is the most devastating complication of intraocular surgery with extremely poor visual prognosis. Old age, hypertension, and atherosclerosis are recognized as systemic risk factors [1]. The ocular risk factors include preexisting glaucoma, ocular inflammation, aphakia, or vitreous loss during surgery.

Spontaneous suprachoroidal haemorrhage on the other hand has been attributed to choroidal and posterior ciliary vasculature fragility [2]. Various risk factors observed in previous reports include coagulation disorders and anticoagulant therapy with deranged INR [3]. Association with ARMD [4] and axial myopia [5] points towards choroidal vasculature abnormalities. The precipitating factors include sudden ocular hypotony, or valsalva manoeuvre [6, 7]. Srikanth and Kumar [8] reported a case of spontaneous suprachoroidal haemorrhage and expulsion of all intraocular contents in a patient with decompensated liver disease, with INR of 2.15.

None of the reported risk factors could be identified in our patient. However a self-limiting episode of Hepatitis A was associated with raised serum bilirubin and liver enzymes. A small hemangioma of liver was an incidental finding.

Suprachoroidal haemorrhage causes forward displacement of the lens-iris diaphragm, resulting in angle closure [4] as in our case. The initial treatment in such case is directed towards angle closure. Systemic and local IOP lowering drugs are used to control IOP. Topical pilocarpine is to be avoided as it pushes lens-iris diaphragm forward. Peripheral iridectomy also is not very effective in the early stages.

Once IOP is controlled, the treatment is directed towards suprachoroidal haemorrhage. Surgical drainage is indicated in cases of lens cornea touch, progressive IOP elevation, and intolerable pain. It is best to defer the surgical drainage for 1-2 weeks [1, 9], till clot lysis is completed. For our patient, surgical management was not required as there was no lens-corneal touch and we were able to control IOP with aggressive medical treatment. Moreover haemorrhage showed resolution at 2 weeks.

Suprachoroidal haemorrhage, both surgical and spontaneous, has poor visual prognosis. Yang et al. [10] followed 5 eyes with spontaneous suprachoroidal haemorrhage, four of which had vision of no light perception at the final follow-up. Chorich et al. [11] reported a case with vision improvement to perception of light from no perception after surgical drainage.

The very fact that our case was not associated with any coagulation disorder or any of the previously described risk factors makes it unique. It is only the second bilateral case of spontaneous suprachoroidal haemorrhage to be reported. In previously reported cases where there was improvement in visual acuity, the pretreatment visual acuity was already good and it showed improvement of 2 snellen lines, while, in our patient, there was drastic improvement of visual acuity from perception of light with inaccurate PR to 6/12.
